# Purity determination of synthetic glucagon using a mass balance approach

**DOI:** 10.1038/s41598-020-61109-9

**Published:** 2020-03-10

**Authors:** Xinxue Wang, Fangyan Zhang, Hongmei Li, Peng Xiao, Fuhai Su, Bei Xu, Wei Sun, Dewei Song

**Affiliations:** 1 National Institute of Metrology, China/Division of Chemical Metrology and Analytical Science, Beijing, China; 2 Beijing University of Chemical Technology, Beijing, China

**Keywords:** Biochemistry, Biological techniques

## Abstract

Due to the widespread use of synthetic peptide drugs, their quantification and the analysis of impurities have become increasingly important in clinical and medical settings. Moreover, quantifying proteins using synthetic peptides as internal or external standards is a general approach, and the key to this approach is the knowing purities of the peptides. In this paper, synthetic glucagon was quantified using a mass balance method. The impurities in glucagon were analyzed and then accurately quantified separately. Karl Fischer (KF) titration and ion chromatography (IC) were used to determine the water and trifluoroacetic acid (TFA) contents in the samples, respectively. Furthermore, the inorganic ion content in the samples was determined by inductively coupled plasma mass spectrometry (ICP-MS). The sequence of peptide impurities was identified by a Thermo Fisher Orbitrap mass. Samples were determined to be 896.36 ± 0.68 mg/g after subtracting all impurity masses from the sample mass. The result can be traced to SI units.

## Introduction

As an advancement in biological and recombinant technologies, peptide drugs have become an important class of clinical drugs because of their efficacy. However, their inherent instability must be addressed in terms of peptide drug use and transport. To overcome these issues, a method that can precisely quantify a drug’s purity is of central importance. Compared with traditional quantification methods such as immunoassay and liquid chromatography - Ultraviolet (LC-UV), the mass balance method is advantageous because of its high precision and the traceability of its results to SI units. As a basic guideline for the establishment of chemical reference standards, the mass balance method has long been recommended by the WHO^1^ and was adopted by the European Pharmacopoeia and International Pharmacopoeia^2,3^. In addition to its ability to determine the content of only the main constituent, the mass balance method can also be used to analyze and quantify impurities in peptide drugs. In general, amino acid dehydration has been used to synthesize peptide drugs, and mistakes such as omission, loss, or increase of the amino acids may occur during this process; peptide decomposition may also create impurities. Although some peptide impurities are similar to the principal component in terms of composition and construction, their chemistries are different. Therefore, it is necessary to identify and quantify peptide impurities. In addition, when their impurities are quantified separately, peptide drugs can be quantified more precisely.

Immunologists perform amino acid analysis by liquid chromatography-isotope dilution mass spectrometry (LC-IDMS) or liquid chromatography-mass spectrometry (LC-MS). Due to their incompatibility with molecules larger than approximately 10 kDa, protein quantification by these methods usually requires proteolytic digestion of large analytes with an enzyme such as trypsin to cleave it into a set of smaller peptides, one of which, the signature peptide, is subsequently used as a surrogate for the protein for quantification^4^. In the above methods, synthetic peptides may be used as internal or external standards to quantify the cleaved peptides. Toth *et al*.^5^ demonstrated the applicability of an integrated immobilized enzyme reactor-liquid chromatography-tandem mass spectrometry (IMER-LC-MS/MS) platform for the quantitation of apolipoproteins in serum using synthetic target peptides as internal standards. Van den Broek *et al*.^6^ evaluated the tryptic digestion efficiency for implementation in quantitative clinical chemistry proteomics using a synthetic stable-isotope-labeled standard. Bronsema *et al*.^7^ summarized the different options for internal standardization in the absolute targeted quantification of protein biopharmaceuticals by LC-MS/MS using synthetic stable-isotope-labeled peptides as internal standards. For these applications, the purity of the synthetic peptides is essential for quantification.

In this paper, we chose synthetic glucagon consisting of 29 amino acids as an example and adopted the mass balance method to determine its purity. This method is a way to accurately quantify sample impurities such as organic and inorganic compounds and moisture, among others, and then deduct the mass of the impurities from the total sample mass. Isotope dilution mass spectrometry (IDMS) was used to quantify the peptide impurities in our sample. IDMS is suitable as a definitive method because it does not depend on the sample recovery, has high precision, and can be tested for bias and unknown interferences^8^. IDMS methods involve adding a labeled version of the analyte as an internal standard to the sample, followed by sample processing and subsequent measurement of the peak area ratio of unlabeled to labeled analyte by LC-MS. IDMS is recognized as a primary method^9–11^ and has been widely used to establish reference measurement procedures for biomarkers such as creatinine^12–14^, glucose^15–17^, cholesterol^18–20^, urea^21,22^, uric acid^23,24^ and triglycerides^8,25^. IDMS is also a highly accurate quantitative method. Compared with common quantitative methods, IDMS has numerous advantages, such as low uncertainty, high precision, high accuracy, and results that can be traced to SI units^26^.

The purity of synthetic glucagon, as determined by LC-UV, is reportedly higher than 98%. However, this purity is inaccurate based on comparison with the result obtained from the mass balance method. Using the mass balance method, we accurately quantified the impurities in synthetic glucagon separately and then determined the glucagon content by subtracting the masses of all impurities from the total sample mass.

## Results

### Concentration of TFA,water and inorganic ions in synthetic glucagon

**Figure 1:**
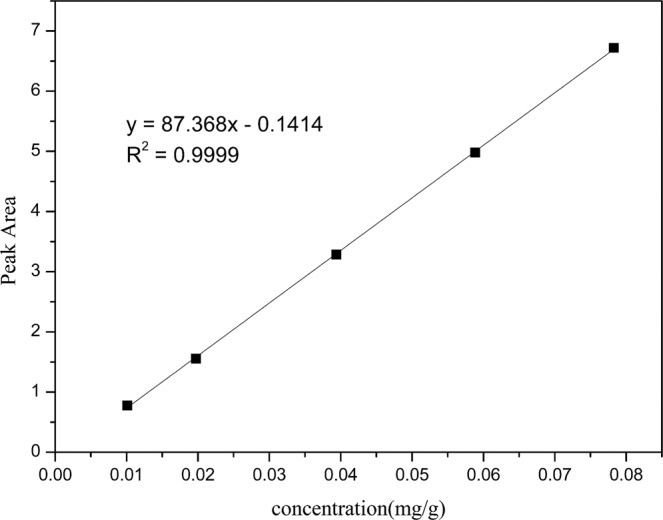
Five-point TFA standard curve.

Five concentrations of pure TFA were used for this analysis, with high linearity (R^2^ = 0.9999) as shown in Fig. 1. The accurately calculated TFA concentration was an approximately intermediate value within the five TFA concentrations in the gradient. After calculation, the content of TFA in the synthetic glucagon was 103.03 mg/g. The results of the TFA contents in the sample are shown in Table 1.

**Table 1:** Concentration of TFA and Water in Synthetic Glucagon.

Sample	TFA(mg/g)	Average (mg/g)	RSD (%)
S1	100.2	103.03	2.49
S2	103.8		
S3	105.1		
**Sample**	**Water(mg/g)**	**Average (mg/g)**	**RSD (%)**
S1	51.0	50.2	1.36
S2	50.2		
S3	49.7		

In this experiment, the Karl Fischer titration method was used to determine the moisture in the sample. The principle is that the Karl Fischer reagent in the electrolysis cell of the instrument is injected into the aqueous sample when the equilibrium is reached, and the water participates in the redox reaction of iodine and sulfur dioxide. In the presence of pyridine and methanol, the iodine consumed by the reaction is again electrolyzed at the anode so that the redox reaction continues until the water is completely consumed. For multiple measurements, the data with a relative standard deviation (RSD) of three consecutive measurements within 3% was taken as the experimental result. The water content was 50.2 mg/g. The results of the water contents in the sample are shown in Table 1.

In this study, Cl, Fe, Si, Ca in synthetic glucagon were detected, the content of all inorganic ions is less than 0.1%, so all can be ignored. The contents of the main inorganic ions in the sample are shown in Table 2.

**Table 2:** Concentrations of the Main Inorganic Ions in Synthetic Glucagon.

Element	Q2	Tuning mode	Concentration	Semiquantitation unit	Semiquantitation factor	Integration time	Internal standard tag
Cl	35	No Gas	25511.335	ppb	220.678	0.02	FALSE
Fe	56	No Gas	5220.198	ppb	100847.577	0.02	FALSE
Si	28	No Gas	3106.212	ppb	7805.727	0.02	FALSE
Ca	43	No Gas	1628.621	ppb	180.584	0.02	FALSE

### Concentration of impurity peptides in synthetic glucagon

The impurities cannot be discriminated by LC-UV because the RTs of the peptide impurities with similar amino acid sequences to that of the main component are close to the RT of the main component. The peaks of impurities with low concentrations in the sample are small and frequently masked by the main component peak. Therefore, the impurities were qualitatively analyzed based on m/z differences by LC-MS in this study. The m/z values of all peptide impurities were different from the m/z values of each other and of the main component. The method was then used for qualitative analysis in an international data comparison. The results of the main peptide impurities in the sample are shown in Table 3. We synthesized nine impurity peptides by their amino acid sequence and confirmed the purity of each synthetic peptide by isotope dilution mass spectrometry, which is a guarantee for accurate quantitation of impurity peptides. We selected three amino acids (Ala, Leu, and Phe) to determine the purity of each peptide. These three amino acids are reference materials, ensuring the accuracy and traceability of the results. Using Leu as an example, the concentration of amino acids after hydrolysis was calculated as follows:

1 $${c}_{Leu}=\frac{P{P}_{H}{m}_{s}[{R}_{sample}({I}_{1}-{I}_{2})-({I}_{1}{R}_{2}-{I}_{2}{R}_{1})]}{m({R}_{1}-{R}_{2})}$$

where P is the purity of Leu, P_H_ is the hydrolysis efficiency of the peptide, m_s_ is the mass of isotopically labeled Leu, R_sample_ is the Leu/isotopically labeled Leu area ratio in the sample solution, I_1_ is the Leu/isotopically labeled Leu mass ratio in the lower-level standard solution, I_2_ is the Leu/isotopically labeled Leu mass ratio in the higher-level standard solution, R_1_ is the Leu/isotopically labeled Leu area ratio in the lower-level standard solution, R_2_ is the Leu/isotopically labeled Leu area ratio in the higher-level standard solution, and m is the sample mass.

**Table 3:** Main Peptide Impurities in Synthetic Glucagon Determined by LC-MS.

Number of amino acids in the peptide	Peptide sequence	MS area	Peptide ratio
14	SRRAQDFVQWLMNT	81172108	0.011903479
28	SQGTFTSDYSKYLDSRRAQDFVQWLMNT	62983626	0.00923623
20	YSKYLDSRRAQDFVQWLMNT	34185324	0.005013105
15	HSQGTFTSDYSKYLD	32850172	0.004817311
28	HSQGTFTSDYSKYLDSRRAQDFVQWLMN	28321355	0.004153183
21	HSQGTFTSDYSKYLDSRRAQD	23206890	0.003403173
23	TSDYSKYLDSRRAQDFVQWLMNT	14260270.5	0.002091196
26	HSQGTFTSDYSKYLDSRRAQDFVQWL	9641092	0.001413817
15	DSRRAQDFVQWLMNT	8312444.5	0.001218978

The mass fraction of the total peptide calculated from Leu (P_Pep_, Leu) was determined as follows:

2 $${P}_{{\rm{Pep}},{\rm{Leu}}}=\frac{c{}_{{\rm{Leu}}}M_{{\rm{Pep}}}}{{N}_{{\rm{Leu}}}{M}_{{\rm{Leu}}}}$$

where M_Pep_ is the molecular weight of the peptide impurity, M_Leu_ is the molecular weight of Leu, and N_Leu_ is the number of Leu residues in the peptide impurity.

The mass fractions of the total peptide calculated for Phe and Ala (P_Pep,Phe_ and P_Pep,Ala_) were obtained using equations similar to the above two equations. Then, the mass fraction of the total peptide was calculated as follows:

3 $${P}_{{\rm{Pep}}}=\frac{{P}_{{\rm{Pep}},{\rm{Leu}}}+{P}_{{\rm{Pep}},{\rm{Phe}}}+{P}_{{\rm{Pep}},{\rm{Ala}}}}{3}$$

According to Eq. (3), the uncertainty in the content of the synthetic peptide impurity can be determined as follows:

4 $${u}_{pep}=\frac{\sqrt{{{u}_{leu}}^{2}+{{u}_{phe}}^{2}+{{u}_{ala}}^{2}}}{3}$$

where u_pep_ is the uncertainty in the peptide impurity content. The numbers 1 to 9 represent the nine peptide impurities, and their purities are shown in Table 4.

**Table 4:** Information of the Nine Peptide Impurities found in Synthetic Glucagon.

	Amino acid sequence	Purity (mg/g)	Expanded uncertainty (k = 2) (g/g)	Content (mg/g)	Uncertainty (mg/g)
1	SRRAQDFVQWLMNT	714.3	15.62	1.06024	1.7985E-05
2	SQGTFTSDYSKYLDSRRAQDFVQWLMNT	758.7	6.46	1.29908	3.5642E-05
3	YSKYLDSRRAQDFVQWLMNT	530.2	13.85	1.31261	9.4552E-05
4	HSQGTFTSDYSDYSKYLD	705.7	18.15	2.25328	3.7186E-05
5	HSQGTFTSDYSKYLDSRRAQDFVQWLMN	774.0	41.43	4.64365	1.6596E-05
6	HSQGTFTSDYSKYLDSRRAQD	740.4	6.16	4.52593	2.0753E-05
7	TSDYSKYLDSRRAQDFVQWLMNT	747.8	14.48	2.48599	3.6043E-05
8	HSQGTFTSDYSKYLDSRRAQDFVQWL	783.1	49.19	9.74943	1.9108E-05
9	DSRRAQDFVQWLMNT	726.8	36.87	1.05215	1.7348E-05

### The mass fraction of synthetic glucagon

Since inorganic impurities can be neglected, the mass fraction of glucagon was determined as follows:

5 $${w}_{glucagon}=1000-{w}_{w}-{w}_{TFA}-\sum {w}_{xi}$$

where W_glucagon_ (mg/g) is the mass fraction of glucagon determined using the mass balance method, W_W_ (0.502 mg/g) is the mass fraction of water in the glucagon sample, W_TFA_ (103.03 mg/g) is the mass fraction of TFA in the glucagon sample, and W_xi_ (0.0112 mg/g) is the mass fraction of peptide impurities in the glucagon sample.

In this way, the content of glucagon in the sample was determined to be 896.36 mg/g.

6 $${u}_{{({\rm{w}}}_{glu})}=\sqrt{{u}_{({w}_{w})}{}^{2}+{u}_{w{(}_{IM})}{}^{2}+{u}_{w{(}_{TFA})}{}^{2}}$$

where ${u}_{{({\rm{w}}}_{glu})}$ is the uncertainty in the glucagon content, ${u}_{({w}_{w})}$ is the uncertainty in the water content, ${u}_{w{(}_{IM})}$ is the uncertainty in the contents of all peptide impurities, and ${u}_{w{(}_{TFA})}$ is the uncertainty in the TFA content. Using Eq. (6), the uncertainty in the glucagon content was determined to be 0.683 mg/g.

The purity of synthetic glucagon reported by the manufacturer is 983.72 mg/g, and the result determined using the mass balance method was 896.36 mg/g, resulting in a difference of 87.36 mg/g. We also analyzed the synthetic glucagon solution by LC-UV and obtained a purity of over 970 mg/g, with small visible impurities. This result is similar to that of the manufacturer. Thus, the result obtained by determining the content of only the main constituent and not the sample impurities using LC-UV is not accurate because some impurities do not absorb ultraviolet radiation, while others may have absorption features that overlap with those of the main constituent. Compared to LC-UV, the mass balance method is an absolute quantitative method that could be traced to SI units.

The Pharmacopeia of US and Europe lists several requirements for impurities in peptide drugs. First, the impurity must be quantified if its content is >1%; second, the impurity should be analyzed qualitatively if its content is >0.5%; and finally, the presence of an impurity must be reported if its content is >0.1%. According to these requirements, the water and TFA reported herein as impurities in synthetic glucagon should be quantified. The reason that such guidelines would require reporting for such small contents of peptide impurities is possibly because the chosen synthetic peptide possesses a small molecular weight and simple structure. Extremely small mass peptide impurities in peptide drugs would have a significant effect on the efficacy, especially for sensitive patients. On the other hand, the determination of very low levels of peptide impurities can also reflect the level of development of proteins and peptides drugs. The peptide impurities that exist in some peptide drugs have high molecular weights and complex structures, which may not be overlooked when using the mass balance method for their quantification.

## Conclusions

We developed a mass balance method to quantify synthetic peptides by accurately determining the content of impurities. Mass balance provides a reference method for the qualitative and quantitative analysis of impurities in peptide drugs and contributes to current research efforts aimed at determining how impurities affect efficacy. The type and content of impurities in peptide drugs are closely related to their production methods, processes, and the structure of the drug itself. This study investigated the impurities of synthetic glucagon by solid-phase method. Generally speaking, the impurities produced under the same process conditions are the similar. This research has good reference significance for the determination of peptide drug impurities in the solid-phase synthesis process production plant. For the quantification of unknown impurities, the structure of the impurities must be determined before quantification, otherwise accurate quantification cannot be achieved under current technical conditions. In addition, in this work, we accurately quantified synthetic peptides that could be used as target peptides for determining protein purity. The bias could be decreased by determining the purity of the synthetic peptide using the mass balance method before quantifying the protein using the synthetic peptide as an internal or external standard.

## Methods

### Experimental

#### Reagents and chemicals

Synthetic glucagon and nine peptide impurities were acquired from GenScript Biological Technology (Nanjing, China). Deionized water was obtained from a Milli-Q Integral system (resistivity = 18.2 MΩ/cm). Three amino acids (phenylalanine (Phe, 99.4%), alanine (Ala, 99.4%), and leucine (Leu, 99.1%)) were purchased from Sigma (Tokyo, Japan), and three labeled amino acids (C- and N-labeled Phe, C- and N-labeled Ala, and C- and N-labeled Leu, each with a purity of 99%) were purchased from Cambridge Isotope Laboratories Inc. (Andover, USA). Acetonitrile was supplied by Merck KGaA (Darmstadt, Germany). Formic acid was supplied by Fisher Scientific (Shanghai, China). Hydrochloric acid was supplied by Beijing Chemical Works (Beijing, China). Karl Fischer (KF) reagents were obtained from Sigma-Aldrich (Shanghai, China). Trifluoroacetic acid (TFA, purity = 99.9%) was obtained from J&K Scientific (Beijing, China). HCl (analytical grade) was purchased from Beijing Chemical Works (Beijing, China).

#### Apparatus

An electrothermostatic blast oven (DHG-9140A) from Yiheng Scientific (Shanghai, China) and a vacuum drying oven (VC 50) from Salvis Vacuucenter (Schweiz, Switzerland) were used for polypeptide chain hydrolysis. An Orbitrap FusionTM LumosTM TribridTM mass spectrometer from Thermo Fisher Scientific was used for sequence identification of peptide impurities. A Dionex™ ICS-5000+ capillary tube high-pressure ion chromatography (HPIC) system from Thermo Fisher Scientific (Waltham, MA, USA) was used for IC. A 6410 triple quadrupole mass spectrometer from Agilent Technologies (Santa Clara, USA) was used for mass spectrometric detection. A Karl Fischer coulometer was obtained from Mettler Toledo (Shanghai, China). An Agilent 1200 instrument was obtained from Agilent Technologies (Santa Clara, USA). An Agilent 8900 inductively coupled plasma mass spectrometer (ICP-MS) (Santa Clara, USA) was used for ICP-MS analysis.

### Determination of TFA in synthetic glucagon

In this study, we used the external standard method to quantify the TFA in the synthetic peptides. First, the pure TFA used in the external standard method was quantified by the nuclear magnetic method. Then, the pure TFA was configured into a series of gradient concentration solutions by accurate weighing and detected by ion chromatography to draw a standard curve. The content of TFA in the synthetic peptide was calculated from the standard curve. The purity of the TFA standard determined by nuclear magnetic resonance spectroscopy was 992 mg/g, which could be traced to SI units (acesulfame potassium reference material as the internal standard). The conditions of 19F-qNMR for determining the purity of TFA are shown below:

Ofloxacin (85~95 mg) was accurately weighed in an airtight bottle; 30 mL of DMSO was added; and 21~29 mg of TFA was injected by syringe into the bottle. After the ofloxacin dissolved, the solution was transferred to an NMR tube. A capillary containing D_2_O was inserted for locking onto the magnetic field.

Instrument: Bruker 400 Ultrashield Plus

Pulse angle: 30 degrees

Relaxation delay: 5 s

Scan number: 128

Offset of pulse: −96.4 ppm

Spectral width: 237.1643 ppm

Size of FID: 65536

Relaxation delay: 5 s

The content of TFA in the sample was quantified using pure TFA having a determined purity. The ion chromatogram of TFA in the sample was compared with that of TFA in a standard solution, which qualitatively confirmed the TFA impurity in synthetic glucagon. We first prepared the sample solution at an appropriate concentration using ultrapure water. A series of TFA standard solutions with a mass concentration gradient was prepared from a TFA stock solution using ultrapure water. The gradient solutions were then detected by IC to yield a standard curve between the concentration and peak area. The TFA standard solution and the sample solution are simultaneously detected by the IC, and then, the TFA of the sample is accurately quantified using the plotted standard curve and the peak area generated by the sample.

Chromatography: columns, Dionex IonPac AG19 anionic guard column (50 mm × 4 mm) and IonPac AS19 anion-exchange separation column (250 mm × 4 mm); column temperature, 30 °C; leachate, 55 mM KOH; isocratic elution, 1.0 mL/min; injection volume, 25 µL; and detector, automatically renewable inhibitory conductance detector (ASRS, 4 mm, and curb., 137 mA).

### Determination of the water in synthetic glucagon

In this experiment, the Karl Fischer titration method was used to determine the moisture in the sample. To avoid the influence of moisture in the air, we determined the amount of water in synthetic glucagon in a glovebox. The humidity of the glovebox was controlled within a range of 1% by aerating with high-purity nitrogen. Three samples placed inside of a glovebox at room temperature for 1.5 h were direct added to a KF reaction vessel. Then, the samples were used for detection under the following conditions: electrolysis rate, “normal”; polarization current, 2 µA; end voltage, 100 mV; and minimum titration time, 180 s. The drift was determined over 180 s. The recovery of the electrode without a diaphragm using Coulomat AG was 104.51% (0.54%). For multiple measurements, the data with a relative standard deviation (RSD) of three consecutive measurements within 3% was taken as the experimental result.

### Determination of the inorganic ions in synthetic glucagon

The inorganic ions in glucagon were determined by inductively coupled plasma mass spectrometry. A certain weight sample was added to tube of 2 ml for ICP-MS. The following ICP-MS parameters were used:

RF power: 750–850 W

Carrier gas flow rate: 0.95 L min-1

Sampling depth: 6 mm

Cones: Nickel

Cell gas flow rate: 2 mL min-1 helium

Extraction lens: −100 V to −125 V

Octopole bias: −16 V

Quadrupole bias: −14 V

Measured isotopes/dwell time: 79Br (0.1 s), 81Br (0.1 s)

Optional plasma gas: N_2_ at 55 psi

### Determination and quantification of peptide impurities in synthetic glucagon

**Figure 2:**
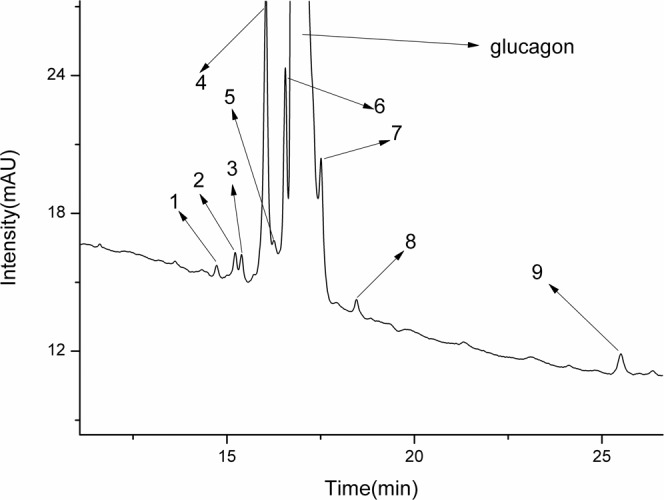
Chromatogram of a synthetic glucagon solution analyzed by LC.

The sample was analyzed by LC-UV, and the resulting chromatogram (Fig. 2) revealed impurities in the sample. Since the differences in the amino acid sequences of the peptide impurities and the main component are very small, their retention times (RTs) are very similar, and all of the peptide impurities cannot be chromatographically separated from the main component. Therefore, we analyzed the sample by LC-MS, which differentiates the impurities by the m/z values. The conditions were used:

Instrument: Agilent 6410 Triple Quard LC/MS

Chromatographic column: Agilent Zorbax SB-Aq 3.5 µm, 2.1 × 150 mm

Mobile phase: A: 0.8 mmol/L perfluorooctanoic acid + 0.05% trifluoroacetic

acid aqueous solution B: acetonitrile

Injection volume: 10 µL

Column temperature: 30 °C

Gradient elution at a flow rate of 0.2 mL/min was set as follows: 1% to 30% B over 5 min, 30% to 40% B over 5 min, 40% to 50% B over 5 min, 50% to 80% B over 1 min, 80% B for 3 min, 80% to 1% B over 3 min, 1% B for 9 min.

The MS1 spectra were analyzed by Protein Discovery software (Thermo Fisher, version1.4). Nine (Table 4) of the peptide impurities that had contents over 0.1% were selected for quantification.

**Figure 3:**
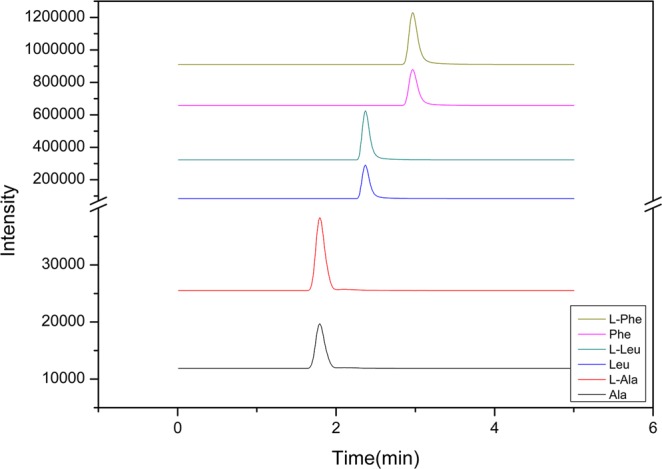
Mass spectra of the amino acids hydrolyzed from the peptide impurities.

Then, we synthesized nine impurity peptides according to their amino acid sequences and then determined the purities of the nine synthesized peptides by amino acid hydrolysis. In the hydrolysis experiment, three amino acids (Ala, Leu, and Phe) were used to determine the purity of each peptide. These three amino acids are all reference materials, which ensure the accuracy and traceability of the analysis results. A solution of the three amino acids of known weight is added to a known weight of synthetic peptide solution, and the resulting solution is hydrolyzed at 110 °C for 24 h. After filtering, the hydrolyzed solution was detected by MS. The three amino acids are well separated and have the same RT as their corresponding C- and N-labeled amino acids. The results are shown in Fig. 3. By calculating the peak area ratio between the amino acids hydrolyzed from the peptide impurities and the amino acids of known weight, the amino acid content in solution is obtained. Then, we determine the purities of the nine peptides used as calibrants for the quantification according to their amino acid sequences. The detection conditions for the three amino acids were as follows: Ala, m/z 90.0 → 44.1; Leu, m/z 132.0 → 86.1; and Phe, m/z 166.0 → 120.0. The detection conditions for the three labeled amino acids were as follows: L-Ala, m/z 94.0 → 47.1; L-Leu, m/z 139.0 → 92.1; and L-Phe, m/z 176.0 → 129.0.

We then quantified the impurity peptides in the sample using synthetic peptides of known purities. A concentration gradient of solutions of each synthetic peptide was prepared, and these solutions were separately subjected to mass spectrometry to obtain a regression curve of the concentration of each impurity peptide. Then, the glucagon sample solution was prepared, and the nine impurity peptides in the sample were separately detected by mass spectrometry. The peak area of each impurity peptide is brought into a regression curve corresponding to the pure peptide to calculate the impurity peptides of the glucagon sample. Since the synthetic impurity peptides are detected by mass spectrometry, the optimal conditions for mass spectrometry need to be determined. Each impurity peptide was configured as a solution, and the condition parameters were optimized by mass spectrometry. The m/z of each peptide was determined by mass spectrometry in the SCAN mode. Using the SIM mode, the Fragmentor of each parent ion is optimized to determine the best Fragmentor. The mass spectrometric conditions for detecting the impurity peptides in the sample are shown in Table 5.

**Table 5:** Mass Spectrometric Condition Information of the Nine Peptide Impurities Found in Synthetic Glucago.

amino acid sequences of nine peptides	m/z	Fragment (V)	Collision energy (V)
SRRAQDFVQWLMNT	584.9 → 578.8	110	8
SQGTFTSDYSKYLDSRRAQDFVQWLMNT	837.4 → 833.0	150	7
YSKYLDSRRAQDFVQWLMNT	631.4 → 626.9	120	8
HSQGTFTSDYSDYSKYLD	583.9 → 577.9	115	9
HSQGTFTSDYSKYLDSRRAQDFVQWLMN	677.3 → 580.5	120	11
HSQGTFTSDYSKYLDSRRAQD	616.7 → 366.7	140	22
TSDYSKYLDSRRAQDFVQWLMNT	707.3 → 702.8	130	10
HSQGTFTSDYSKYLDSRRAQDFVQWL	628.3 → 505.7	130	10
DSRRAQDFVQWLMNT	623.3 → 617.4	120	9

## Data Availability

The datasets generated during and/or analyzed during the current study are available from the corresponding author on reasonable request.
